# Conjugated equine estrogen used in postmenopausal women associated with a higher risk of stroke than estradiol

**DOI:** 10.1038/s41598-021-90357-6

**Published:** 2021-05-24

**Authors:** Wei-Chuan Chang, Jen-Hung Wang, Dah-Ching Ding

**Affiliations:** 1 Department of Medical Research, Hualien Tzu Chi Hospital, Buddhist Tzu Chi Foundation, and Tzu Chi University, Hualien, Taiwan; 2 Department of Obstetrics and Gynecology, Hualien Tzu Chi Hospital, Buddhist Tzu Chi General Hospital, Buddhist Tzu Chi Foundation, and Tzu Chi University, No. 707, Chung-Yang Rd., Sec. 3, Hualien, Taiwan, ROC; 3 Department of Obstetrics and Gynecology, College of Medicine, Tzu Chi University, Hualien, Taiwan

**Keywords:** Cardiology, Health care, Medical research

## Abstract

This study aimed to evaluate the risk of ischemic stroke (IS) in hormone therapy (HT) with oral conjugated equine estrogen (CEE) and estradiol (E2) in postmenopausal women in Taiwan. A retrospective cohort study was conducted using the Taiwan National Health Insurance Research Database, a population-based healthcare claims dataset. Eligible women, aged 40–65 years, who received HT with E2 and CEE orally were enrolled. The primary outcome was IS. Propensity score matching with menopausal age and comorbidities was used. Cox proportional hazard regression models were used to calculate the incidence and hazard ratios (HRs) for IS. The mean menopausal ages of the E2 and CEE groups were 50.31 ± 4.99 and 50.45 ± 5.31 years, respectively. After adjusting for age and comorbidities, the incidence of IS was 1.17-fold higher in the women treated with CEE than in those treated with E2 (4.24 vs. 3.61/1000 person-years), with an adjusted HR (aHR) of 1.23 (95% confidence interval [CI] 1.05–1.44). Moreover, HT with CEE initiated within 5 years of menopause had a higher HR than E2 (aHR = 1.20; 95% CI 1.02–1.42). In conclusion, HT with oral CEE might be associated with a higher risk of IS than E2 in postmenopausal Taiwanese women. The use of HT with CEE should be cautioned with the risk of IS.

## Introduction

Stroke is a condition caused by vascular disease affecting the brain, which causes a persistent neurologic deficit^1^. The incidence of stroke is estimated at 94 and 117 per 100,000 person-years in high-income and other countries, respectively^2^. Stroke is still the leading cause of disability and third leading cause of death for women^3^. The risk factors for stroke include smoking, hypertension, unhealthy diet, overweight, less physical activity, diabetes mellitus (DM), hyperlipidemia, and others^4^.

In postmenopausal women with menopausal symptoms, hormone therapy (HT) is used to increase life quality^5^. Several forms of HT such as locally applied (cream, pessary, or ring) and systemically applied (oral use, transdermal patches, or implants) can be provided^6^. Hormone use can be classified as estrogen alone, estrogen plus progestin, gonadomimetics such as tibolone, and selective estrogen receptor modulators^7^. The time schedules for hormone use include daily use of estrogen, sequential use with progestin for 2 weeks, and continuous use combined with progestin. The types of estrogen can be classified as 17-beta-estradiol (E2), conjugated equine estrogen (CEE), and ethinyl estradiol. Contrastingly, progestin (synthetic progesterone) can be classified as generation indicates when they go to the market. Norethindrone acetate (NE) and medroxyprogesterone acetate (MPA) are first-generation progestins and the main progestins used in HT. In Taiwan, the most common HT is CEE at 0.625 mg or E2 at 2 mg combined with 5 mg MPA or 1 mg NE in patients with an intact uterus^8^. Estrogen alone is prescribed for patients without a uterus^8^. Alternatively, selective estrogen receptor modulators are used for the prevention and treatment of osteoporosis^9^. The time to use HT was suggested as near menopause or before the age of 60 years^10^.

CEE comprises equine estrogens, estrone, equilin, and equilenin, each conjugated to a sulfate group different from E2^11^. CEE and E2 have been reported to act on tryptophan hydroxylase-2 (related to cognitive and affective disorders) of different brain locations^12^. E2 has been found to increase cognition and decrease anxiety and depression more effectively than CEE^12^. The previous study showed that a lower dose of CEE would increase estrone, but not E2, and did not have any protective effect. In contrast, a higher dose of CEE would increase both the estrone and E2 levels^13^. They concluded that an increase in estrone, but not E2 level, might interfere with the cognitive function^14^. Moreover, one study reported that E2 provided a higher serum E2 level than the same dose of CEE, which provided a higher estrone level^15^. This difference may reflect the different compositions of hormone drugs.

The 2002 Women’s Health Initiative (WHI) study revealed that HT with CEE caused several side effects, including coronary heart disease, stroke, breast cancer, and pulmonary embolism^16,17^. Furthermore, our previous study showed that HT in Taiwan was associated with an increased risk of ischemic stroke (IS) and venous thromboembolism (VTE)^18^. Possible mechanisms of HT caused several side effects that may result from the formation of fibrin fibers, hemostasis imbalance, a preexisting endothelial injury, or an estrogen receptor-mediated dysfunction^19,20^. However, E2 has been considered to be beneficial for stroke due to its neuroprotective action in the animal study^11^. The previous case–control study showed that a low E2 level and high polygenic risk scores were associated with the risk of IS^21^. Previous studies showed lower risks of cardiovascular events in postmenopausal women taking oral E2 or esterified estrogen than in those taking CEE^22,23^.

Nevertheless, HT with CEE is associated with the risk of stroke^24^. We assumed that different forms of estrogen might be associated with different risks of stroke.

This study aimed to determine the risk of IS in menopausal women receiving HT with different forms of estrogen (CEE vs. E2) in Taiwan based on a high-quality national health database.

## Materials and methods

### Data source

We used the longitudinal health insurance database 2000 (LHID2000), which comprised all medical claim data from 2000 to 2016. The LHID2000 was randomly sampled by 2 million beneficiaries from the registry of all National Health Insurance enrollees for the year 2000, and maintained by the Taiwan Health and Welfare Data Science Center of the Ministry of Health and Welfare. The database contained data on demographic characteristics, medical records, medications, and disease diagnoses. Diseases were identified using ICD-9-CM and ICD-10-CM. This study was approved by the Research Ethics Committee of Hualien Tzu Chi Hospital (IRB107-179-C). Informed consent was waived due to low risk by the Research Ethics Committee of Hualien Tzu Chi Hospital. We confirm that all methods were performed in accordance with the relevant guidelines and regulations.

### Study design

**Figure 1:**
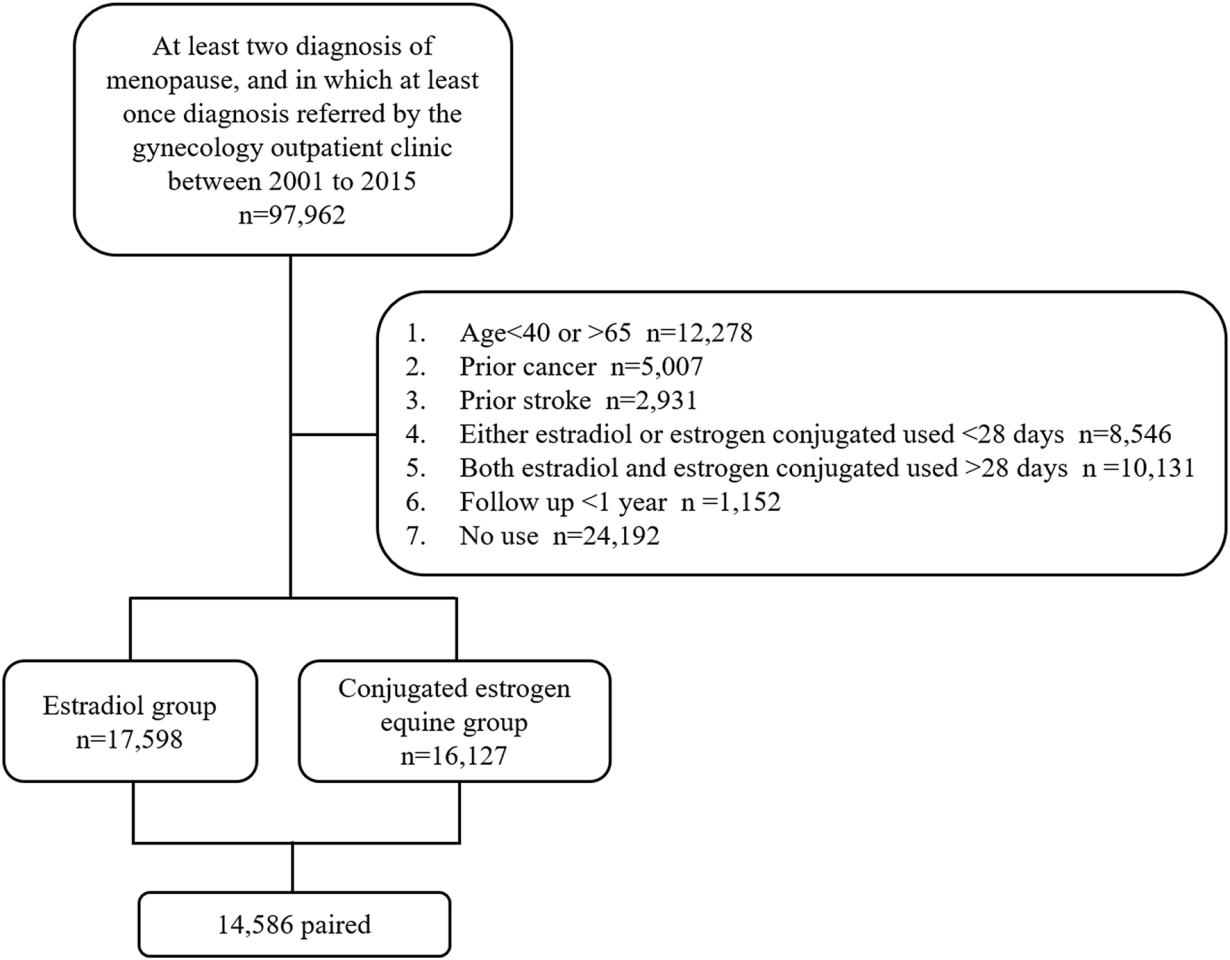
Study flow chart.

A retrospective population-based cohort study was performed to examine the association between HT among menopausal women (ICD-9-CM code 627) treated with E2 or CEE and IS. Women within the menopausal ages of 45–65 years at first diagnosis, who continually took E2 or CEE for over 28 days, were enrolled in the study. Both inpatient and outpatient data were included. We excluded women who had a prior diagnosis of malignancy (ICD-9-CM codes 140–208) or stroke (ICD-9-CM codes 430–438; ICD-10-CM codes I60–I69) before index date; with a follow-up period from index date to endpoint of less than 1 year; and who took both E2 and CEE for over 28 days. We performed propensity score matching by menopause age and comorbidities listed in Table 1 to deal with potential confounder between the two groups. Finally, 14,586 paired women were eligible for the study cohort from 2001 to 2015 (Fig. 1).

**Table 1:** Characteristic of the estradiol and conjugated equine estrogen groups.

Characteristic	Estradiol (n = 14,586)	CEE (n = 14,586)	p-value	SMD
**Menopause age, years**	50.31 ± 4.99	50.45 ± 5.31	0.018	0.039
**Comorbidities**				
Hypertension	7807 (53.52%)	7772 (53.28%)	0.681	0.004
Diabetes mellitus	3821 (26.20%)	3781 (25.92%)	0.594	0.007
Hyperlipidemia	9093 (62.34%)	8982 (61.58%)	0.181	0.014
Angina	2156 (14.78%)	2156 (14.78%)	1.000	< 0.001
COPD	3994 (27.38%)	3966 (27.19%)	0.713	0.004
Heart failure	311 (2.13%)	304 (2.08%)	0.775	< 0.001
CAD	4811 (32.98%)	4770 (32.70%)	0.609	0.006
AF	244 (1.67%)	234 (1.60%)	0.645	0.008
**Duration of HT, days**	158.3 ± 281.8	163.1 ± 269.7	0.137	0.017
< 0.5 years	11,516 (78.95%)	11,251 (77.14%)	< 0.001	0.044
≥ 0.5 years	3070 (21.05%)	3335 (22.86%)		
Hysterectomy	942 (6.46%)	2720 (18.65%)	< 0.001	0.374
**Estrogen**				
Low	6478 (44.41%)	202 (1.38%)		
Intermediate	8108 (55.59%)	14,233 (97.58%)		
High	0 (0.0%)	151 (1.04%)		
**Progestin**	13,892 (95.24%)	9888 (67.79%)	< 0.001	0.754
Low	5204 (37.46%)	2948 (29.81%)		
Intermediate	7306 (52.59%)	6369 (64.41%)		
High	1382 (9.95%)	571 (5.77%)		
**MPA**	7080 (48.55%)	9833 (67.41%)	< 0.001	0.388
Low	3086 (43.59%)	2958 (30.08%)		
Intermediate	2778 (38.24%)	6393 (65.02%)		
High	1216 (17.17%)	482 (4.90%)		
**NE**	9875 (67.70%)	237 (1.62%)	< 0.001	1.931
Low	3887 (39.36%)	68 (28.69%)		
Intermediate	5787 (58.60%)	76 (32.07%)		
High	201 (2.04%)	93 (39.24%)		
**HT initiation time from menopause**	1.87 ± 2.79	0.87 ± 1.75	< 0.001	0.425
≤ 5 years	12,552 (86.06%)	13,928 (95.49%)	< 0.001	0.330
> 5 years	2034 (13.94%)	658 (4.51%)		

Data are shown as means ± standard deviations or proportion of the characteristics.

Dose: low, 1 mg/day of E2, 0.3 mg/day of CEE, 2.5 mg/day of MPA, and 0.5 mg/day of NE; intermediate, 2 mg/day of E2, 0.625 mg/day of CEE, 5 mg/day of MPA, and 1 mg/day of NE; high, 3 mg/day of E2, 1.25 mg/day of CEE, 10 mg/day of MPA, and 2 mg/day of NE.

CEE, conjugated equine estrogen; SMD, standardized mean difference; COPD, chronic obstructive pulmonary disease; CAD, coronary artery disease; AF, atrial fibrillation; HT, hormone therapy; MPA, medroxyprogesterone acetate; NE, norethindrone acetate.

### Population and covariates

We divided the study cohort into two groups to explore the different risks of IS (ICD-9-CM codes 433, 434, and 436; ICD-10-CM codes I63, I65, and I66) between E2 and CEE. If women continually took E2 only for over 28 days during the follow-up period, we included them in the E2 group. The same classification rule was applied for the CEE group. The index date was the first date of taking HT continually. The endpoint was the occurrence of IS (December 31, 2016). Baseline comorbidities were ascertained at 1 year before the index date. Comorbidities included hypertension (ICD-9-CM codes 401–405), diabetes mellitus (DM, ICD-9-CM code 250), hyperlipidemia (ICD-9-CM code 272), angina (ICD-9-CM code 413), chronic obstructive pulmonary disease (COPD, ICD-9-CM codes 491, 492, and 496), heart failure (ICD-9-CM codes 571.2, 571.5, and 571.6), coronary artery disease (CAD, ICD-9-CM codes 410–414), and atrial fibrillation (AF, ICD-9-CM code 427.3). Further, we divided estrogen and progestin into different doses to compare dose response effect (low, 1 mg/day of E2, 0.3 mg/day of CEE, 2.5 mg/day of MPA, and 0.5 mg/day of NE; intermediate, 2 mg/day of E2, 0.625 mg/day of CEE, 5 mg/day of MPA, and 1 mg/day of NE; high, 3 mg/day of E2, 1.25 mg/day of CEE, 10 mg/day of MPA, and 2 mg/day of NE).

### Statistical analyses

We used an independent sample t-test for continuous variables and the Chi-square test for categorical variables. The SMD was calculated to compare baseline characteristics between the two groups. We calculated the HRs and 95% CIs for IS in the Cox proportional hazards model. All models were adjusted for all characteristics listed in Table 1. Interaction tests were performed for all comorbidity subgroups. Statistical analysis was performed with SAS version 9.4 for Windows (SAS Institute, Inc., Cary, NC), and a statistical chart was created using Stata version 15 for windows (LP. 2013; StataCorp, College Station, TX). Statistical significance was set at two-sided p < 0.05 or SMD > 0.1.

## Results

### Subject characteristics

The baseline characteristics of the two groups are shown in Table 1. The percentage of hysterectomy in the CEE group was significantly higher than that in the E2 group (standardized mean difference [SMD] = 0.374). The percentage of progestin use in the E2 group was higher than the CEE group (standardized mean difference [SMD] = 0.754). Moreover, the type of progestin was significantly different between the two groups. The percentage of MPA use was higher in the CEE group (standardized mean difference [SMD] = 0.388). In contrast, the percentage of NE use was higher in the E2 group (standardized mean difference [SMD] = 1.931). HT initiation time from menopause was longer in the E2 group than in the CEE group (SMD = 0.425). Menopause age, comorbidities, and duration of HT between the two groups were not significantly different.

### Risk of ischemic stroke

Table 2 shows the incidence and HR of IS in patients who received HT with CEE compared with that of those who received E2. After follow-up, the incidence of IS was 1.17-fold higher in women treated with CEE than in those treated with E2 (4.24 vs. 3.61/1000 person-years), with an adjusted HR (aHR) of 1.23 (95% confidence interval [CI] 1.05–1.44).

**Table 2:** Hazard ratios for ischemic stroke in the estradiol and conjugated equine estrogen groups.

Group	IS, n (%)	PY	IR	Crude HR (95%CI)	Adjusted HR (95%CI)
Estradiol	406 (2.78%)	53,411.4	3.61	1	1
CEE	705 (4.83%)	84,914.1	4.24	1.12 (0.99–1.27)	1.23 (1.05–1.44)

Cox regression model was adjusted for age, comorbidities, and other characteristics listed in Table 1.

IS, ischemic stroke; PY, person-year; IR, incidence rate; HR, hazard ratio; CEE, conjugated equine estrogen.

### Risk of ischemic stroke in different doses of estrogen and progestin

In Table 3, different doses of estrogen and progestin did not affect the occurrence of IS.

**Table 3:** Hazard ratios for ischemic stroke in different doses of estradiol and conjugated equine estrogen groups.

Group	IS, n (%)	PY	IR	Crude HR (95%CI)	Adjusted HR (95%CI)
**Estradiol**					
Low	148 (2.28%)	43,105.2	3.43	1	1
Intermediate	258 (3.18%)	69,349.7	3.72	1.04 (0.84–1.27)	1.08 (0.74–1.48)
High	–	–	–	–	–
**CEE**					
Low	14 (6.93%)	2636.1	5.31	1	1
Intermediate	684 (4.81%)	161,940.6	4.22	0.81 (0.48–1.38)	0.83 (0.41–1.67)
High	7 (4.64%)	1747.5	4.01	0.76 (0.31–1.89)	1.41 (0.46–4.33)
**Progestin**					
Low	296 (3.63%)	70,812.5	4.18	1	1
Intermediate	556 (4.07%)	136,784.8	4.07	0.96 (0.83–1.11)	0.92 (0.79–1.08)
High	45 (2.30%)	17,011.2	2.65	0.63 (0.47–0.87)	0.76 (0.55–1.06)
**MPA**					
Low	218 (3.61%)	53,968.2	4.04	1	1
Intermediate	368 (4.01%)	95,509.9	3.85	0.94 (0.79–1.11)	1.00 (0.84–1.18)
High	40 (2.36%)	15,140.5	2.64	0.65 (0.47–0.92)	0.76 (0.55–1.06)
**NE**					
Low	112 (2.83%)	31,154.8	3.60	1	1
Intermediate	226 (3.85%)	53,164.7	4.25	1.17 (0.93–1.46)	1.73 (0.91–3.29)
High	6 (2.04%)	2215.2	2.71	0.76 (0.34–1.73)	1.62 (0.67–3.96)

Cox regression model was adjusted for age and comorbidities listed in Table 1.

HR, hazard ratio; PY, person-year; CI, confidence interval; CEE, conjugated equine estrogen; MPA, medroxyprogesterone acetate; NE, norethindrone acetate.

### Stratified analysis of the risk of ischemic stroke with comorbidities

The incidence and HR of IS in patients with CEE stratified by comorbidities are shown in Table 4. In subgroup analysis, we found that the CEE group had a higher risk of IS than the E2 group (with hypertension, DM, hyperlipidemia, angina, COPD, and CAD, without heart failure and AF), although a few of the comparisons did not reach statistical significance. The tests for subgroups in the analyses of the comorbidities and HT drugs revealed no interaction effect (Table 4).

**Table 4:** Subgroup analysis for ischemic stroke risk between the conjugated equine estrogen and estradiol groups.

Characteristics	aHR (95%CI)	p for interaction
**Hypertension**		
No	1.22 (0.82–1.81)	0.556
Yes	1.23 (1.04–1.47)	
**Diabetes mellitus**		
No	1.14 (0.91–1.42)	0.328
Yes	1.33 (1.06–1.66)	
**Hyperlipidemia**		
No	0.93 (0.61–1.42)	0.119
Yes	1.29 (1.09–1.53)	
**Angina**		
No	1.18 (0.98–1.42)	0.820
Yes	1.35 (1.00–1.82)	
**COPD**		
No	1.19 (0.97–1.46)	0.752
Yes	1.29 (1.00–1.09)	
**Heart failure**		
No	1.24 (1.06–1.46)	0.184
Yes	0.79 (0.31–2.04)	
**CAD**		
No	1.16 (0.91–1.48)	0.991
Yes	1.28 (1.04–1.57)	
**AF**		
No	1.21 (1.02–1.42)	0.429
Yes	1.54 (0.88–2.71)	
**Duration of HT**		
< 0.5 years	1.17 (0.97–1.40)	0.561
0.5–1 years	1.32 (0.95–1.82)	
**HT initiation time from menopause**		
≤ 5 years	1.20 (1.02–1.42)	0.081
> 5 years	1.77 (0.87–3.59)	

Cox regression model was adjusted for characteristics listed in Table 1.

HR, hazard ratio; COPD, chronic obstructive pulmonary disease; CAD, coronary artery disease; AF, atrial fibrillation; HT, hormone therapy.

### Subgroup analysis of the risk of ischemic stroke with HT duration and initiation time from menopause

The duration of HT with CEE (< 0.5 years or 0.5–1 years) was not associated with a higher risk of IS than that of HT with E2 (Table 4). Additionally, HT with CEE initiated within 5 years of menopause had a higher HR than HT with E2 (HR = 1.20; 95% CI 1.02–1.42) (Table 4).

## Discussion

This population cohort evaluated 14,586 and 14,586 women who received E2 and CEE, respectively. The mean menopausal ages were 50.31 and 50.45 years in the E2 and CEE groups, respectively. The CEE group had a 1.23-fold higher risk of IS than the E2 group. HT with CEE initiated within 5 years of menopause had a higher HR of IS than HT with E2.

A recent comprehensive review and meta-analysis regarding the effects of HT on cardiovascular disease, including stroke, showed that HT increased the risk of VTE (randomized controlled trials, summary estimate (SE), 1.70; 95% CI 1.33–2.16; observational studies, SE, 1.32; 95% CI 1.13–1.54) and stroke (SE, 1.14; 95% CI 1.04–1.25)^25^. As regards underlying disease, HT in patients with underlying diseases was associated with a higher risk of stroke (SE, 1.14; 95% CI 1.04–1.26). Regarding the regimen type, combined HT was associated with a higher risk of stroke (SE, 1.14; 85% CI 1.01–1.29). With regard to the duration of HT, HT for a duration of 5 years or more was associated with a higher risk of stroke (SE, 1.13; 95% CI 1.03–1.25).

Low estrone levels in postmenopausal women are reportedly associated with a high risk for coronary artery disease (CAD)^26^. In an animal study, CEE alone was neuroprotective in the cortex but not in the subcortical brain regions^27^. Furthermore, a previous study showed that obese older women receiving HT with E2 had the highest estrone levels, which may be associated with thromboembolic events, compared with non-obese, younger women^28^. They suggested that HT should be cautioned for aged and obese postmenopausal women. A higher IS risk associated with the use of CEE alone than with the use of esterified estrogen was reported^29^. A previous study concluded that CEE was associated with a higher risk of atrial fibrillation than E2^30^. The use of E2 did not reduce the risk of reinfarction in postmenopausal women with one previous myocardial infarction^31^. Moreover, E2 did not reduce the recurrence risk of stroke in postmenopausal women^32^. Previous studies have shown lower risks of cardiovascular events in postmenopausal women taking oral E2 or esterified estrogen than in those taking CEE^22,23^. Smith et al. reported an increased risk of stroke with oral CEE than with E2 (odds ratio [OR], 1.13; 95% CI 0.55–2.31; p = 0.74)^22^. In our study, we found that CEE was associated with a higher risk of stroke than E2, which was comparable to previous studies.

The previous study compared CEE to esterified estrogen and found that duration of CEE use is associated with the risk of stroke^29^. In addition, the previous meta-analysis showed that HT duration of 5 years or more was associated with an increased risk of IS^25^. The mean duration of using HT was 2.1–3.9 years. A previous Swedish study showed that an HT (CEE or E2) duration less or more than 5 years was not associated with the risk of stroke^33^. Forty percent of enrolled patients used HT for more than 5 years^33^. In our study, the average duration of use was extremely short, as most women (80%) used HT for less than 6 months and about 20% used for more than 6 months. Therefore, we set the HT duration at < 0.5 years and 0.5–1 year. In our study, the duration of HT with CEE was not associated with a higher risk of stroke than that of HT with E2. The result may be due to the short duration of HT compared with that in other studies.

A previous study showed that increased stroke risk was associated with CEE in the 50–59 years group (HR, 1.09) compared with that in the 60–69 years (HR, 1.72) and 70–79 years groups (HR, 1.52). However, after statistical analyses, the effect of age was not significant^24^. Moreover, CEE by years since menopause or years since bilateral oophorectomy had no effect^24^.

Another study revealed that initiating HT early within 5 years of menopause was associated with decreased risk of stroke compared with when never used^33^. However, another study showed an increasing twofold risk of IS after the first 6 months of HT^34^. HT initiation after more than 5 years of menopause was associated with an increased risk of stroke when CEE was used as a single therapy^33^. The previous meta-analysis indicated that late HT (age ≥ 60 years or initiation after 10 years since menopause) is associated with a higher risk of stroke (SE, 1.17; 95% CI 1.01–1.37) in randomized trials^25^. However, the same meta-analysis failed to demonstrate the same conclusion in the observational study^25^. In our study, we showed that HT with CEE initiated within 5 years of menopause was associated with a higher risk of stroke than HT with E2. The cause of difference between studies may be due to different study settings and ethnic groups. Moreover, in our study, most women received HT within 5 years, which may have affected the outcome of IS.

The mechanism of CEE causing increased stroke incidence is not well known. Previous studies reported that CEE has longer-effect metabolites than transdermal E2 and may cause inflammation, whereas E2 is not proinflammatory^35^. Inflammation may cause occluded and hypoperfused vessels, which predisposes to stroke^36^. Therefore, CEE may increase inflammation to cause stroke. Additionally, oxidative stress and autophagy activities may be associated with the pathophysiology IS. E2 is reported to inhibit autophagy and oxidative stress to decrease neurologic deficit after IS^37^. Nevertheless, the detailed mechanisms of stroke in postmenopausal women using CEE or E2 should be further explored.

A previous study showed that estrogen with or without progestin was not associated with the risk of IS (OR, 0.97; 95% CI 0.69–1.37; and OR, 0.94; 95% CI 0.72–1.23, respectively) compared with no use^34^. However, another study showed that estrogen with or without progesterone was associated with an increased risk of cardiovascular disease or stroke (relative risk, 1.35; 95% CI 1.08–1.68; and relative risk, 1.45; 95% CI 1.10–1.92, respectively)^38^. Regarding progestins, the previous study showed that higher progestin dose was associated with an increased risk of IS when compared with never using HT^39^. Regarding types of progestin, cyclic combined use of MPA and NE was associated with an increased risk of IS (RR, 1.12; 95% CI 0.94–1.33; and RR, 1.22; 95% CI 1.10–1.35, respectively)^39^. Further, continuous combined use of NE was associated with an increased risk of IS (RR, 1.38; 95% CI 1.28–1.48)^39^. Another study compared different types of progestogens and found that norpregnane derivatives (nomegestrol acetate) had higher risks of IS (OR, 2.25; 95% CI 1.05–4.81)^40^. In our study, we found that 91–92% of patients received estrogen therapy combined with progestogen. After adjustment, CEE was still associated with a higher risk of IS than E2. Nevertheless, in our study, the type and dosage of progesterone was not associated with the risk of IS.

The dose of estrogen could contribute to stroke risk differently, and a high dose is associated with a higher risk of IS^39^. The study showed that low-dose estrogen was not associated with the risk of IS (RR, 0.87; 95% CI 0.73–1.04); however, high-dose estrogen was associated with IS risk (RR, 1.78; 95% CI 1.42–2.24)^39^. Moreover, another study showed an increased oral estrogen dose associated with a higher risk of IS (intermediate dose, OR, 1.84; 95% CI 1.02–3.30; high dose, OR, 2.41; 95% CI 1.43–4.07)^40^. Nevertheless, in our study, the higher dose of estrogen was not associated with a higher risk of IS.

The strength of the present study is that first, we used the database covering 2 million people sampled from 23 million people in Taiwan. This allowed us to precisely evaluate the association between HT (CEE or E2) and the risk of IS. Second, we used a cohort study, which is the most suitable mode for detecting the association between HT and IS. Third, all medical records can be accurately followed through the National Health Insurance Research Database (NHIRD). It allowed us to follow up on the IS outcome among patients using HT. Finally, we matched the comparison cohort with age, which was the main confounding factor.

Nevertheless, this study had limitations. First, due to the NHIRD comprising data covering only 17 years, the patients had a relatively short follow-up time (6.05 years in the E2 group and 9.81 years in the CEE group). Second, only the oral intake of CEE or E2 was included in this study; other forms of intake, such as dermal or vaginal, and other hormones, such as progesterone, were not considered. Third, body mass index data was not available in the database, which is a significant risk factor for stroke. Further, our database could not be obtained by lifestyle and behavioral factors (e.g., diet and exercise), which could be significant confounders. In our cohort study, we used a propensity score matching which may cause the modification of artifactual effect and decrease the capability of adjusting for confounding factors^41^. Lastly, we used the International Classification of Diseases, 9th Revision, Clinical Modification (ICD-9-CM) code to find comorbidities for cardiovascular risk factors that may not always be accurate^42^. Therefore, these results are moderately statistically precise and should be interpreted with caution.

In conclusion, in postmenopausal Taiwanese women, HT with CEE may be associated with a higher risk of IS than HE with E2. The use of HT with CEE should be cautioned with the risk of IS. Further investigation of this population is warranted.

## Data Availability

All related data was presented in the manuscript.
